# Procedural circulatory support-enabled intracoronary cell delivery in advanced dilated cardiomyopathy

**DOI:** 10.1016/j.isci.2026.117543

**Published:** 2026-09-23

**Authors:** Rohini Ramaseshan, Mohsin Hussain, Dhanuka Perera, Stephen Hamshere, Matthew Kelham, Pedro Pinto, James R. Whiteford, Thomas C. Moseley, Alice Reid, Sonia Bastos, Jessry Russell, Mervyn Andiapen, Lucinda Wynne, Ceri Davies, Thomas Keeble, Samer Fawaz, John Martin, Cono Ariti, Daniel A. Jones, Anthony Mathur

**Affiliations:** 1 Centre for Cardiovascular Medicine and Devices, William Harvey Research Institute, Queen Mary University of London, London, UK; 2 Department of Cardiology, Barts Heart Centre, Barts Health NHS Trust, London, UK; 3 NIHR Barts Biomedical Research Centre, Queen Mary University of London, Charterhouse Square, London EC1M 6BQ, UK; 4 Essex Cardiothoracic Centre, Basildon, UK; 5 Oxon Epidemiology, Calle del Dr. Fleming, 51, Chamartín, 28036 Madrid, Spain; 6 University College London, London, UK; 7 MTRC, Anglia Ruskin School of Medicine, Chelmsford, UK; 8 Genes and Health, Queen Mary University of London, London, UK; 9 University Hospitals Dorset NHS Foundation Trust, Bournemouth, UK; 10 William Harvey Research Institute, Barts and The London School of Medicine and Dentistry, Queen Mary University of London, London, UK

**Keywords:** cell therapy, circulatory support, clinical trial, dilated cardiomyopathy, heart failure

## Abstract

Advanced heart failure due to dilated cardiomyopathy (DCM) has limited treatment options. Although intracoronary autologous cell therapy may improve cardiac function, its use in patients with severe left ventricular dysfunction is limited by procedural risk. This exploratory study investigated whether temporary mechanical circulatory support with an Impella device could enable safer therapy delivery. Twenty patients with DCM and a left ventricular ejection fraction (LVEF) of ≤35% received granulocyte colony-stimulating factor pretreatment followed by Impella-supported intracoronary infusion of autologous bone marrow mononuclear cells. The procedure was feasible, well tolerated, and not associated with major complications. After three months, mean LVEF increased by 2.9%, with numerically greater gains in those with more severe baseline impairment. Participants also experienced symptomatic, exercise capacity, and quality of life improvements. Changes in microvascular function and circulating soluble receptor for advanced glycation end-products (sRAGE) suggested potential mechanistic pathways. As a single-arm study, these findings are hypothesis generating and support further evaluation in randomized controlled trials.

## Highlights

• First trial to use circulatory support to facilitate intracoronary cell delivery
• Symptomatic improvement observed despite no significant LV function benefit
• Feasibility for the combination of mechanical support with biologics

## Graphical abstract

**Figure:**
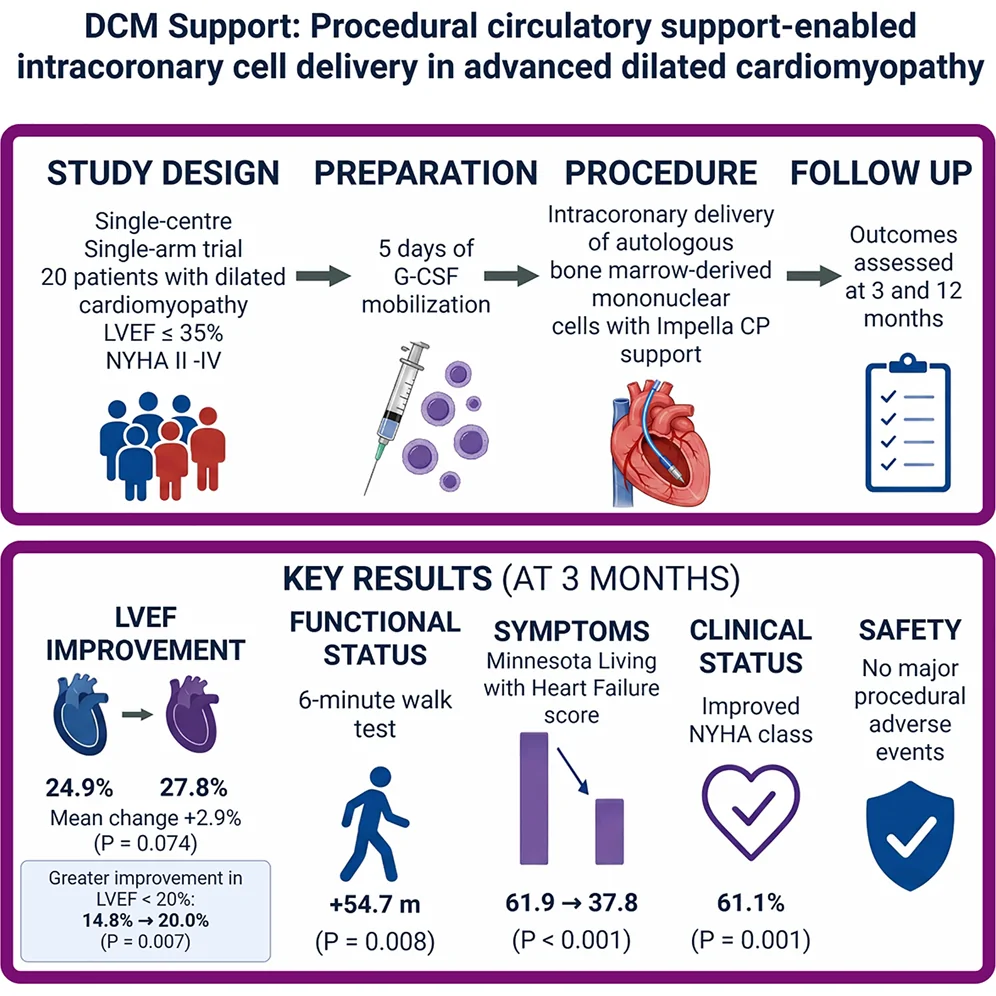

## Teaser

Health sciences; Medicine; Cardiovascular medicine

## Introduction

Estimates regarding the prevalence of dilated cardiomyopathy (DCM) range between 1:250 and 500 in the population across Europe and the US.^1^ Patients with DCM and severe left ventricular systolic dysfunction (>50% have a left ventricular ejection fraction [LVEF] of <35%^2^) remain highly symptomatic despite contemporary guideline-directed therapy and have limited options short of advanced heart-failure interventions.

Previously, cell therapy trials, especially those that used adjunctive cytokine therapy, have demonstrated overall positive results in cardiac function and heart failure symptoms.^3^^,^^4^^,^^5^^,^^6^^,^^7^^,^^8^^,^^9^^,^^10^^,^^11^^,^^12^ Notably, meta-analyses suggest that patients with the greatest degree of systolic impairment may derive the largest therapeutic benefit.^13^^,^^14^^,^^15^ Autologous bone marrow mononuclear cell (BMMNC) therapy has been evaluated in several non-ischemic DCM trials, including REGENERATE-DCM, which demonstrated improvements in functional status, natriuretic peptide levels, and myocardial perfusion.^3^^,^^4^^,^^8^^,^^10^^,^^11^^,^^12^ Allogeneic mesenchymal precursor cell therapy has also been studied in conjunction with durable left ventricular assist device (LVAD) support, with evidence of enhanced myocardial viability and *neo* angiogenesis, highlighting the potential synergy between mechanical unloading and biologic delivery.^16^^,^^17^ These prior observations provide mechanistic support for combining temporary circulatory support with autologous cell therapy for patients with advanced non-ischemic cardiomyopathy.

DCM Support (NCT03572660) was designed as a feasibility study to assess whether short-term hemodynamic support with Impella CP could support safe intracoronary delivery of autologous BMMNCs in patients with severe DCM (LVEF ≤35%). The study also incorporated mechanistic endpoints, including invasive microvascular assessment and inflammatory cytokine profiling, to explore physiological responses to both unloading and cell infusion.

The study was designed as an exploratory feasibility trial focused on safety, procedural delivery, and mechanistic signal generation in a population where invasive approaches are associated with increased hemodynamic risk.

Here, we report the clinical, functional, and mechanistic outcomes from DCM Support.

## Results

### Participant cohort

Between January 2019 and November 2024, 198 patients were screened from specialized heart failure clinics at referring centers across the UK. The trial was paused from March 2020 to the end of May 2021 due to the Covid-19 pandemic. Screening and recruitment of patients resumed in June 2021 once the clinical services became operational again. The participant pathway and eligibility criteria can be found in Figure 1 and Table 1. A CONSORT diagram for the trial is depicted in Figure 2.

**Figure 1:**
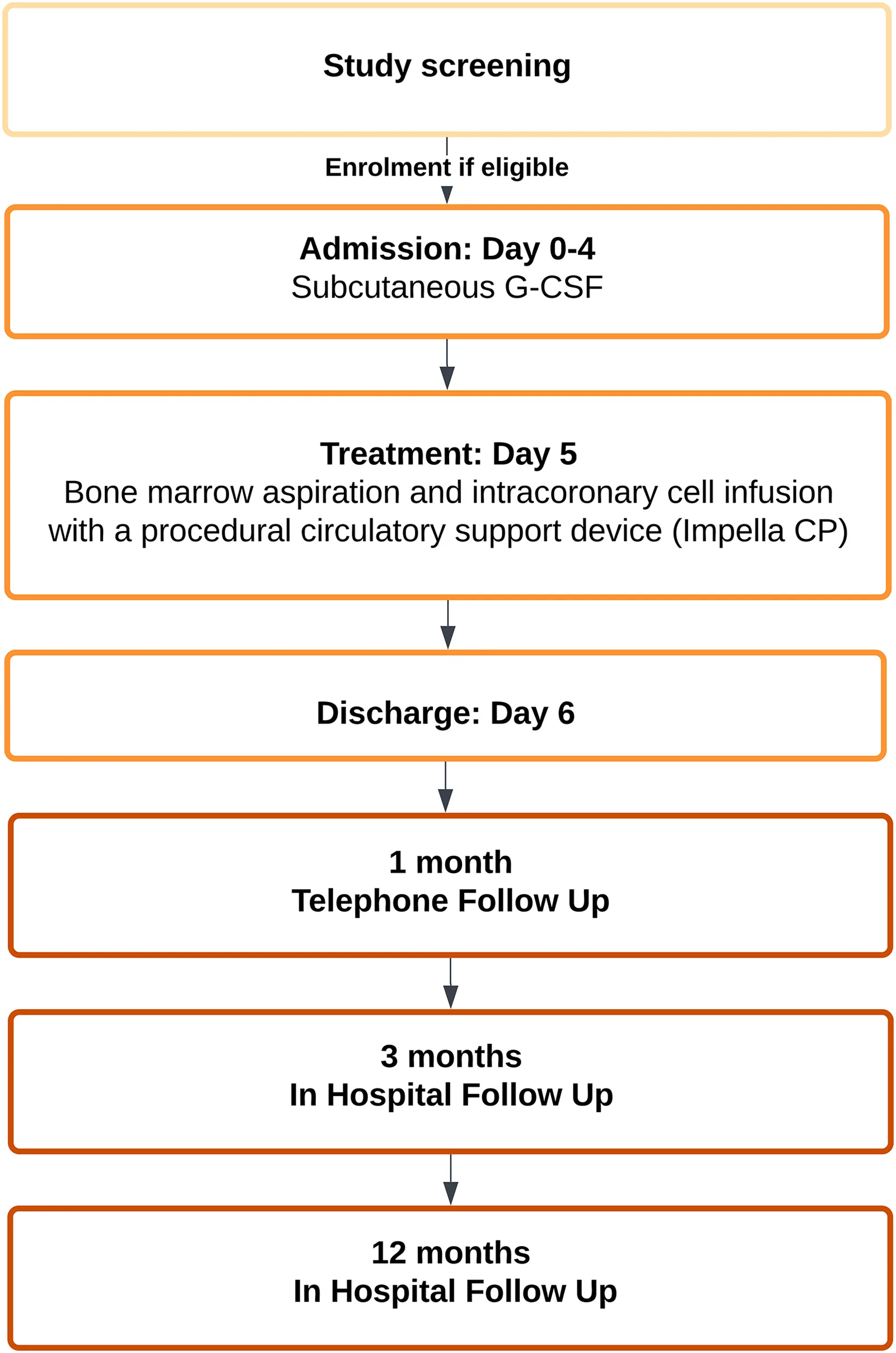
DCM Support study flow G-CSF, granulocyte colony-stimulating factor.

**Table 1:** Eligibility criteria for DCM Support

**Inclusion criteria**

• Patients with a confirmed diagnosis of dilated cardiomyopathy under the supervision of a physician or a heart failure nurse specialist. • NYHA class ≥2 symptoms despite having received optimal medical therapy and appropriate device therapy, as per clinical guidelines for an interval of at least 3 months. • No other treatment options available as part of the current best standard of care. • LVEF ≤35% on any imaging modality performed as part of the screening phase. • Patient between 18 and 85 years old.

**Exclusion criteria**

• Congenital heart disease. • Clinically significant valvular heart disease. • Patients who are not suitable for a Percutaneous Mechanical Support Device (E.g. unsuitable femoral artery anatomy, unable able to lie flat for prolonged time to accommodate the stem cell infusion & presence of LV thrombus) • Weight of patient that exceeds the maximum limit of the cardiac catheterization laboratory table/CT scanner. • Cardiomyopathy 2° to a reversible cause that has not been treated e.g., thyroid disease, alcohol abuse, hypophosphataemia, hypocalcaemia, cocaine abuse, selenium toxicity, & chronic uncontrolled tachycardia. • Cardiomyopathy in association with a neuromuscular disorder e.g., Duchenne’s progressive muscular dystrophy. • Previous cardiac surgery. • Contra-indication for bone marrow aspiration (thrombocytopenia—platelet count <80 × 10(9)/L or extensive surgical scarring/anatomical deformity at site of bone marrow puncture). • Known active infection on admission as defined by a temperature >37.5°C or on a short course of antibiotics. • An active infection of hepatitis B, hepatitis C, syphilis, or HTLV • Known HIV infection • Chronic inflammatory disease requiring on-going medication. • Concomitant disease with a life expectancy of less than one year • Follow-up impossible (no fixed abode, etc.) • Neoplastic disease without documented remission within the past 5 years. • Patients on renal replacement therapy. • Subjects of childbearing potential unless βHCG negative and are on adequate contraception during the trial • Patients falling into the vulnerable category or lacking capacity • Patients who are unable to understand or read written English will be excluded from the trial. • Killip Class III or above

βHCG, beta-human chorionic gonadotrophin; CT, computed tomography; HIV, human immunodeficiency virus; HTLV, human T-lymphotropic virus; LVEF, left ventricular ejection fraction; NYHA, New York Heart Association Class.

**Figure 2:**
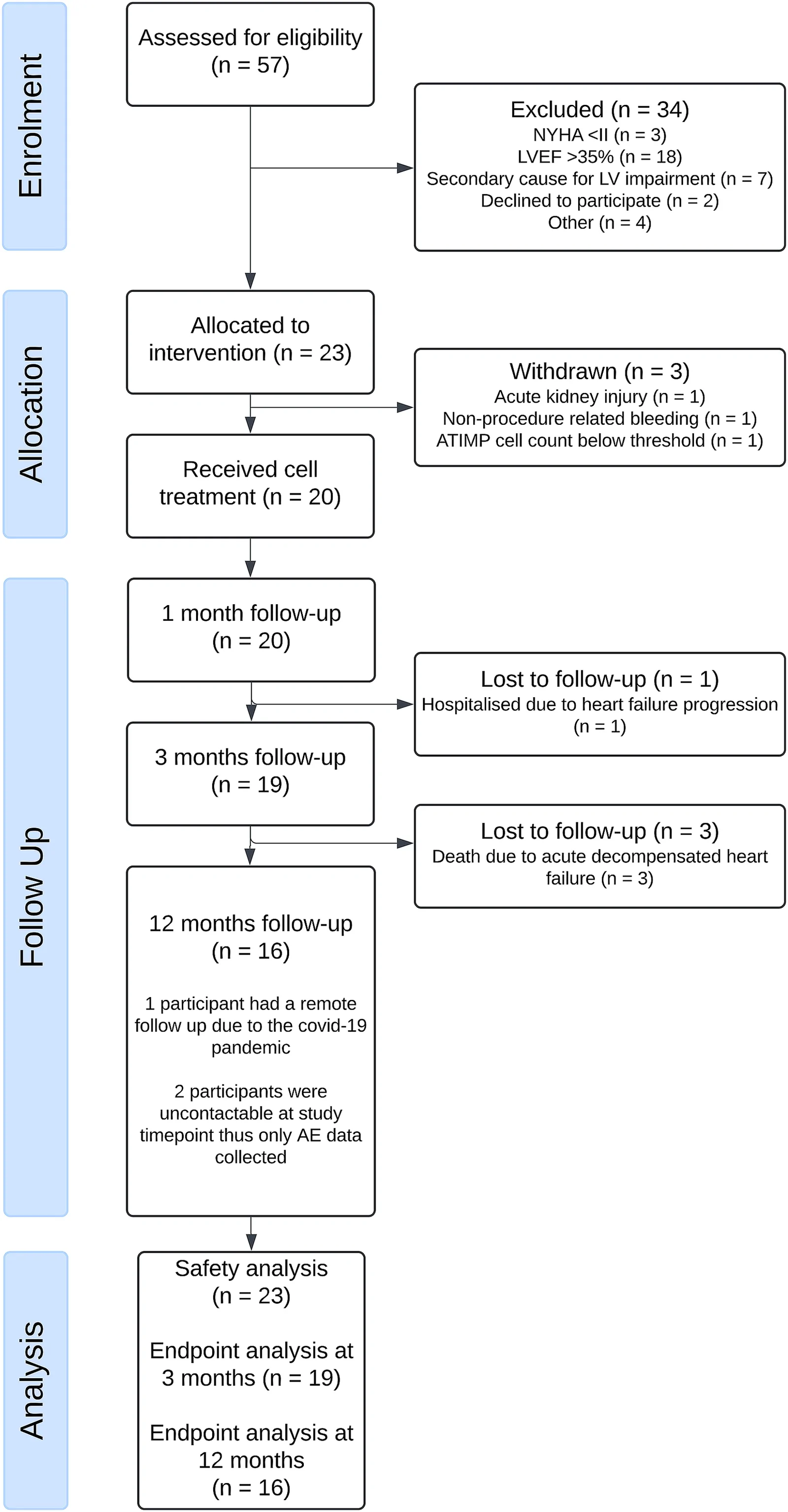
DCM Support consort diagram ATIMP, advanced therapy medicinal product; NYHA, New York Heart Association class.

Of the 198 screened patients, 57 were identified as potentially suitable and provided written informed consent to undergo a screening visit. Thirty-four patients were excluded for the following reasons: baseline New York Heart Association [NYHA] classification <2 (*n* = 3), LVEF >35% on referral echocardiogram (*n* = 18), there was a secondary cause for LV impairment (*n* = 7), they declined to participate (*n* = 2), and other reasons (*n* = 4). Three further participants were withdrawn from the study following enrollment prior to cell administration due to deterioration of renal function, non-procedure related bleeding, and the cellular product’s cell count not meeting threshold. These three participants were replaced in order to reach the 20 participants required for the trial. The full baseline demographics of enrolled patients is presented in Table 2. Of the 19 patients that achieved the primary endpoint, the mean age was 54 ± 13.8 years, 42.1% were female, 78.9% were white British and the mean baseline LVEF was 24.9% ± 10.1%. DCM etiology was: idiopathic (*n* = 9, 47.4%), peripartum (*n* = 4, 21.1%), genetic (*n* = 3, 15.8%), and chemotherapy induced (*n* = 3, 15.8%). Unfortunately, detailed genetic testing was not available in this patient cohort. Changes in medications were minimal during the follow-up period as the inclusion criteria stipulated that the patient had to be on optimal medical therapy as per clinical guidelines for at least 3 months prior to enrollment.

**Table 2:** Baseline characteristics

Baseline characteristics	Analysis population		All subjects	
	(*N* = 19)		(*N* = 23)	
Age-years (mean [SD])	54.0	(13.8)	56.0	(13.7)

**Sex**				

Male	11	(57.9)	12	(52.2)
Female	8	(42.1)	11	(47.8)

**Ethnicity**				

White British	15	(78.9)	16	(69.6)
South Asian	1	(5.3)	2	(8.7)
Asian Other	1	(5.3)	1	(4.3)
Black Caribbean	1	(5.3)	1	(4.3)
Black Other	1	(5.3)	2	(8.7)
Other	0	(0.0)	1	(4.3)

**Dilated cardiomyopathy (DCM) history**				

Time from DCM diagnosis (months)	109.8	(57.4)	117.8	(63.8)
Etiology of DCM				
Chemotherapy	3	(15.8)	3	(13.0)
Genetic	3	(15.8)	5	(21.7)
Idiopathic	9	(47.4)	11	(47.8)
Peripartum	4	(21.1)	4	(17.4)

**Blood pressure**				

Systolic blood pressure (mmHg)	111.2	(12.9)	109.9	(13.2)
Diastolic blood pressure (mmHg)	72.2	(10.3)	71.7	(10.4)

**Smoking status**				

Current smoker	1	(5.3)	1	(4.3)
Ex-smoker	6	(31.6)	7	(30.4)
Never smoked	12	(63.2)	15	(65.2)

**Hospitalisations for heart failure previous 12 months**				

0	14	(73.7)	17	(73.9)
1	2	(10.5)	2	(8.7)
2	1	(5.3)	1	(4.3)
3	0	(0.0)	1	(4.3)
4	2	(10.5)	2	(8.7)

**Comorbidities**				

Previous stroke	1	(5.3)	2	(8.7)
Diabetes	6	(31.6)	8	(34.8)
Hypertension	3	(15.8)	4	(17.4)
Renal Disease	5	(26.3)	8	(34.8)
High Cholesterol	3	(15.8)	3	(13.0)
Atrial fibrillation	7	(36.8)	9	(39.1)
Asthma/COPD	3	(15.8)	4	(17.4)

**Concomitant medications**				

ACEi/ARB	4	(21.1)	7	(30.4)
ARNI + ARB	15	(78.9)	16	(69.6)
Beta Blocker	18	(94.7)	21	(91.3)
Aldosterone antagonists	16	(84.2)	20	(87.0)
SGLT-2i	13	(68.4)	14	(60.9)
Diuretics	15	(78.9)	19	(82.6)
Warfarin	3	(15.8)	4	(17.4)
Direct oral anticoagulants	7	(36.8)	9	(39.1)
Ivabradine	5	(26.3)	6	(26.1)
Digoxin	4	(21.1)	4	(17.4)

**Device type**				

No device	5	(26.3)	5	(21.7)
ICD	8	(42.1)	10	(43.5)
CRT-P	2	(10.5)	2	(8.7)
CRT-D	4	(21.1)	6	(26.1)

Numbers are mean (SD) or frequency (%).

ACEi, angiotensin-converting enzyme inhibitors; ARB, angiotensin II receptor blockers; ARNI, angiotensin receptor neprilysin inhibitor; COPD, chronic obstructive pulmonary disease; CRT-D, cardiac resynchronization therapy defibrillator; CRT-P, cardiac resynchronization therapy pacemaker; ICD, implantable cardioverter-defibrillator; SD, standard deviation; SGLT-2i, sodium-glucose cotransporter-2 inhibitors.

### G-CSF, cell product, and cell infusion data

In response to granulocyte colony-stimulating factor (G-CSF) therapy, peripheral blood total white cell count increased from 7.5 ± 1.9 × 10^9^ on admission (*n* = 22) to 33.8 ± 9.3 × 10^9^ on day 6 (*n* = 20; the day after the cell infusion procedure). The mean value of the total mononuclear cell count in the injected cell product was 298.9 ± 102.5 × 10^6^, and the mean percentage of viable cells was 96.9%. CD34^+^ enumeration of the cell product was undertaken in 11 participants with a mean value of 4 ± 3.9 × 10^6^. More detailed cell phenotyping could not be undertaken in this study due to technical limitations. Vessel selection for cell infusion was at the operator’s discretion based on left or right-sided dominance. Cells were infused into all three coronary arteries (left anterior descending, left circumflex, and right coronary artery) in 4 participants, into two coronary arteries in 15 participants and into one coronary artery in 1 participant. The median duration of circulatory support provided by the Impella was 40 min (interquartile range [IQR] 12.75 min). Based on the physiologist’s reports, the mean heart rate prior to Impella activation was 75 bpm (range 59–130 bpm) and the mean heart rate post-Impella reaching steady state output was 79 bpm (range 59–125 bpm).

### Primary endpoint—Cardiac function

Of the 20 participants that underwent cell treatment, 19 underwent the primary endpoint assessment of LVEF by computed tomography (CT) at 3 months. Thirteen participants showed an improvement in LVEF from baseline (an overall 2.9% increase in global LVEF from 24.9% ± 10.1% at baseline to 27.8% ± 10.4% at 3 months [95% confidence interval [CI] −0.3, 6.1]); however, this did not reach statistical significance (*p* = 0.074; Figure 3). Similarly, although left ventricular end systolic volume (LVESV), left ventricular end diastolic volume (LVEDV), stroke volume, and left ventricular mass increased from baseline to 3 months, none reached statistical significance (Table S1). In the subset of participants with a baseline LVEF <20% (*n* = 8), there was a greater improvement in LVEF of 5.2% (14.8% ± 3% to 20% ± 5.5%, *p* = 0.007). Change in LVEF did not correlate to the total infused BMMNC count (*n* = 19, *p* = 0.285, Table S2) or the CD34^+^ count (*n* = 11, *p* = 0.092, Table S2). In a pre-specified sub-group analysis, participants that were >50 years old, female, had never smoked, and who had pre-procedural troponin <20 ng/L showed a greater improvement in change in LVEF at 3 months, but this did not reach statistical significance (Table S5).

**Figure 3:**
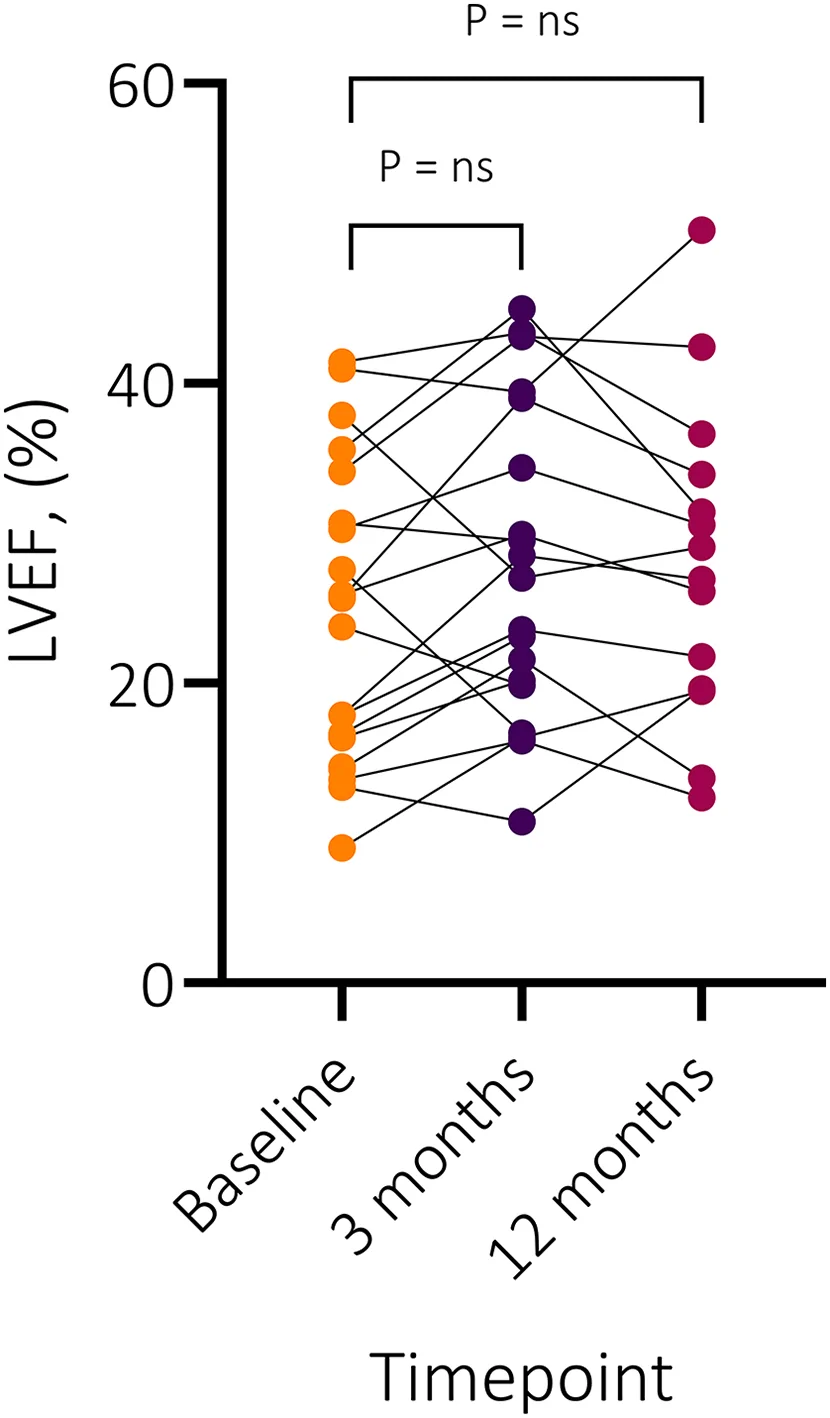
Scatterplot of left ventricular ejection fraction at baseline, 3 months, and 12 months as measured by CT There was a +2.9% increase in LVEF from 24.9% ± 10.1% at baseline to 27.8% ± 10.4% at 3 months (95% CI −0.3, 6.1; *p* = 0.074, *n* = 19). There was an increase of +2.6% from baseline to 12 months (25.5% ± 11.3% to 28.2% ± 10.6%, *p* = 0.136, *n* = 14). LVEF, left ventricular ejection fraction.

At 12 months, although there was a small improvement in LVEF of 2.6% in the overall trial population (*n* = 14, 25.5% ± 11.3% to 28.2% ± 10.6%, *p* = 0.136, Figure 3), 10 out of the 14 participants that reached the 12 month endpoint showed a decrease LVEF between 3 and 12 months. In the subset of participants with a baseline LVEF <20% (*n* = 6), again, there was a greater improvement seen of 4.7% (14.3% ± 3.3% to 19% ± 5.3%, *p* = 0.065).

At 3 months, the mean LVEDV showed an increase of 7.4 mL (328.8–336.2 mL) while the mean LVESV decreased by 1.5 mL (250.3–248.3 mL) (Table S1). At 12 months compared to baseline, the mean LVEDV decreased by 7.4 mL (307.5–300.1 mL), and the mean LVESV decreased by 14.6 mL (233.4–218.8 mL). These changes are consistent with the small improvements seen in ejection fraction. None of these changes reached statistical significance.

### Secondary endpoints

#### Safety

The safety population for the study consisted of all participants who were administered the first dose of G-CSF. Regarding in-hospital procedural safety, there were no deaths, peri-procedural myocardial infarction (as per the SCAI definition), or serious arrythmias (as defined by ventricular tachycardia or ventricular fibrillation). There were no complications (as defined by any event that requires specific treatment and/or prolongation of hospital stay) with G-CSF injection, bone marrow aspiration, intracoronary infusion, or diagnostic coronary angiography.

Fourteen participants had procedure-related adverse events; these were predominantly site pain or bruising due to the bone marrow aspiration or angioplasty/vascular access. There was 1 complication post-procedure related to the mechanical support device access site (a femoral artery pseudoaneurysm) which was managed conservatively.

#### Major adverse cardiac events

At 3 months, 3 participants had major adverse cardiac events (MACE). One participant was hospitalized for acute decompensated heart failure 1 month post-treatment, and two other participants were hospitalized for acute decompensated heart failure 3 months post-treatment. At 3 months, there was no incidence of stroke (defined as clinical presentation including transient ischemic attack), and there were no significant changes in renal function (as measured by creatinine levels) or C-reactive protein. Cumulatively, by 12 months, 5 participants had MACE events. Five participants had heart failure hospitalizations, of which 3 died due to acute decompensated heart failure (at 4 months, 11 months, and 13 months post-treatment). At 12 months there was no incidence of stroke nor changes in renal function or C-reaction protein.

#### NYHA class

At 3 months, there was an improvement in NYHA class in 61.1% of participants (*n* = 18, *p* = 0.001). Four participants (22.2%) had a 2-class improvement, 7 participants (38.8%) had a 1-class improvement, 7 participants (38.8%) had no change, and no participant had a worsened class (Figure 4A). At 12 months, there was an improvement in NYHA class in 66.7% of participants (*n* = 15, *p* = 0.002). Four participants (26.7%) had a 2-class improvement, 6 participants (40%) had a 1-class improvement, 5 participants (33.3%) had no change, and no participant had a worsened NYHA class compared to baseline (Figure 4B).

**Figure 4:**
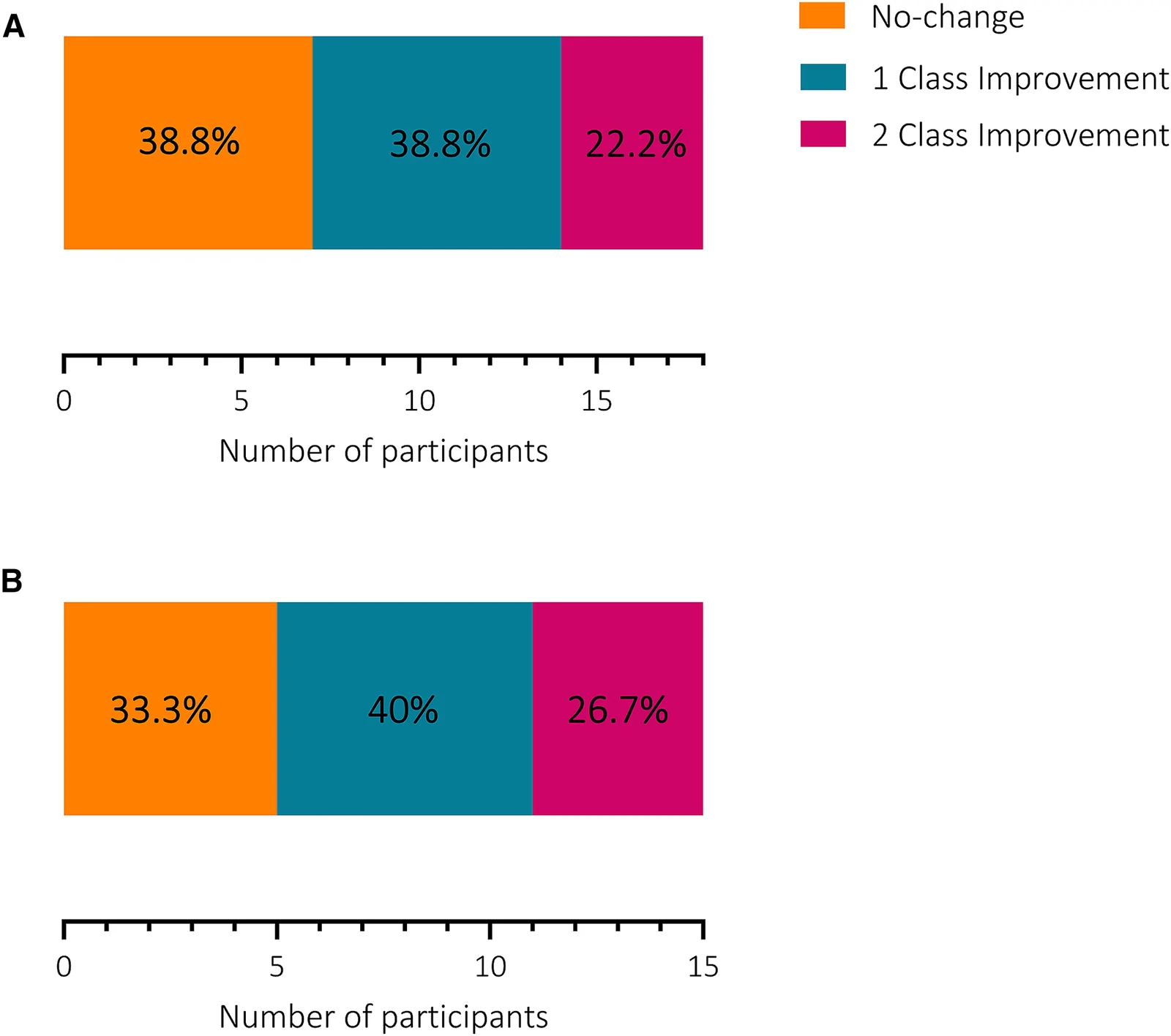
Bar chart showing change in NYHA classification at 3 months and 12 months from baseline (A) At 3 months, 61.1% had an improvement (22.2% reported a 2-class improvement and 38.8% reported a 1-class improvement) and 38.8% had no change. No participant had a worsened NYHA class (*p* = 0.001, *n* = 18). (B) At 12 months, 66.7% had an improvement (26.7% reported a 2 class improvement, and 40% had a 1 class improvement) and 33.3% had no change. No participant had a worsened NYHA class compared to baseline (*n* = 15, *p* = 0.002). NYHA, New York Heart Association.

#### N-terminal pro B-type natriuretic peptide levels

At 3 months, there was slight decrease in N-terminal pro B-type natriuretic peptide levels (NT-proBNP) from baseline (*n* = 18, median 764.5 – 717.5 pg/mL, IQR: 3,864 and 2,520, *p* = 0.13, Figure 5). In the subset of participants with a baseline LVEF <20%, there was a greater improvement (*n* = 7, median 6,089 – 2,708 pg/mL, IQR: 13,413 and 5,611, *p* = 0.04).

**Figure 5:**
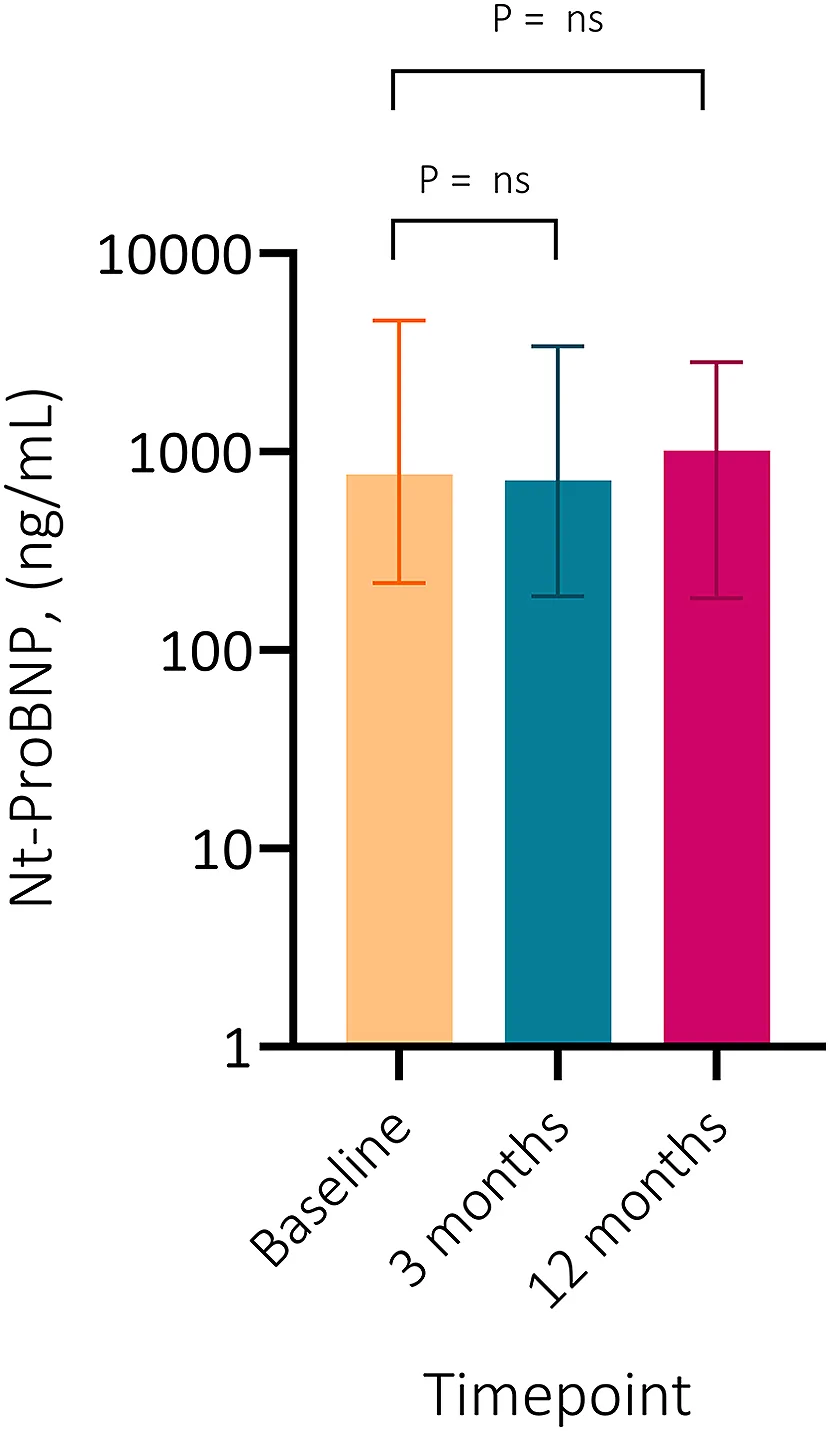
Box and whisker plot showing median NT-proBNP levels at baseline, 3 months and 12 months with error bars for interquartile range At 3 months, there was slight decrease from baseline (median 764.5 – 717.5 pg/mL, IQR 3,864 and 2,520, *p* = 0.13, *n* = 18). At 12 months, there was a small increase from baseline (median 764.5 – 1011.0 pg/mL, IQR 3864.0 and 2393.0, *p* = 0.706, *n* = 15). IQR, interquartile range; NT-proBNP, N-terminal pro B-type natriuretic peptide levels.

At 12 months, there was a small increase from baseline (*n* = 15; median 764.5 –1,011.0 pg/mL, IQR: 3864.0 and 2393.0, *p* = 0.706, Figure 5).

#### Exercise capacity

At 3 months, there was an improvement of +54 m in 6 minute walking test (6MWT) distance compared to baseline (*n* = 18, 280.0 ± 108.6 m to 334.7 ± 131.3 m; *p* = 0.008, Figure 6). These improvements were maintained at 12 months with a +52.6 m increase when compared to baseline (*n* = 14, 297.0 ± 98.9 m to 379.6 ± 111.5 m, *p* = 0.003, Figure 6).

**Figure 6:**
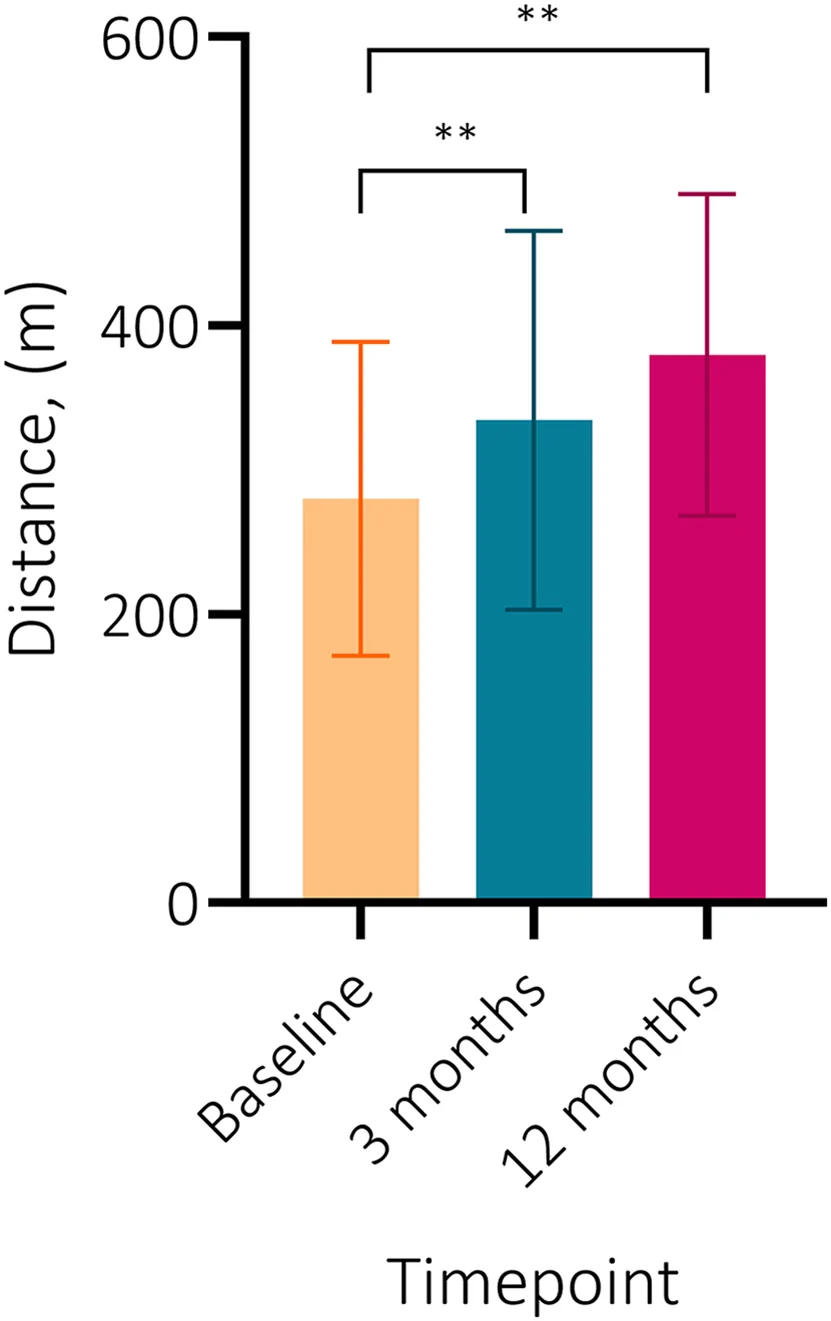
Bar chart showing mean 6 minute walk test distance at baseline, 3 months and 12 months At 3 months, there was a +54m improvement compared to baseline (280.0m ± 108.6m to 334.7m ± 131.3m, *p* = 0.008, *n* = 18). At 12 months, there was a +52.6m increase compared to baseline (297.0m ± 98.9m to 379.6m ± 111.5m, *p* = 0.003, *n* = 14). *p* values are noted as ∗*p* < 0.05; ∗∗*p* < 0.01, error bars show standard deviation.

#### Patient-reported quality of life questionnaire

The mean EQ-5D-5L Time to Trade off (EQ-5D-TTO) index scores were 0.7 ± 0.2 at baseline and 0.7 ± 0.2 at 3 months (*n* = 18, *p* = 0.312; Figure 7). At 12 months, there was no change in EQ-5D TTO scores (*n* = 15, mean scores were 0.7 ± 0.2 at baseline and 0.7 ± 0.2 at 12 months, *p* = 0.192). The EQ-5D-5L Visual Analog Scale (EQ-5D VAS) scores were 53.2 ± 18.2 at baseline and 61.7 ± 15.1 at 3 months (*n* = 18, *p* = 0.056; Figure 8). At 12 months, the EQ-5D VAS scores were 57.5 ± 16.3 at baseline and 66.2 ± 20.6 at 12 months (*n* = 15, *p* = 0.251, Figure 8).

**Figure 7:**
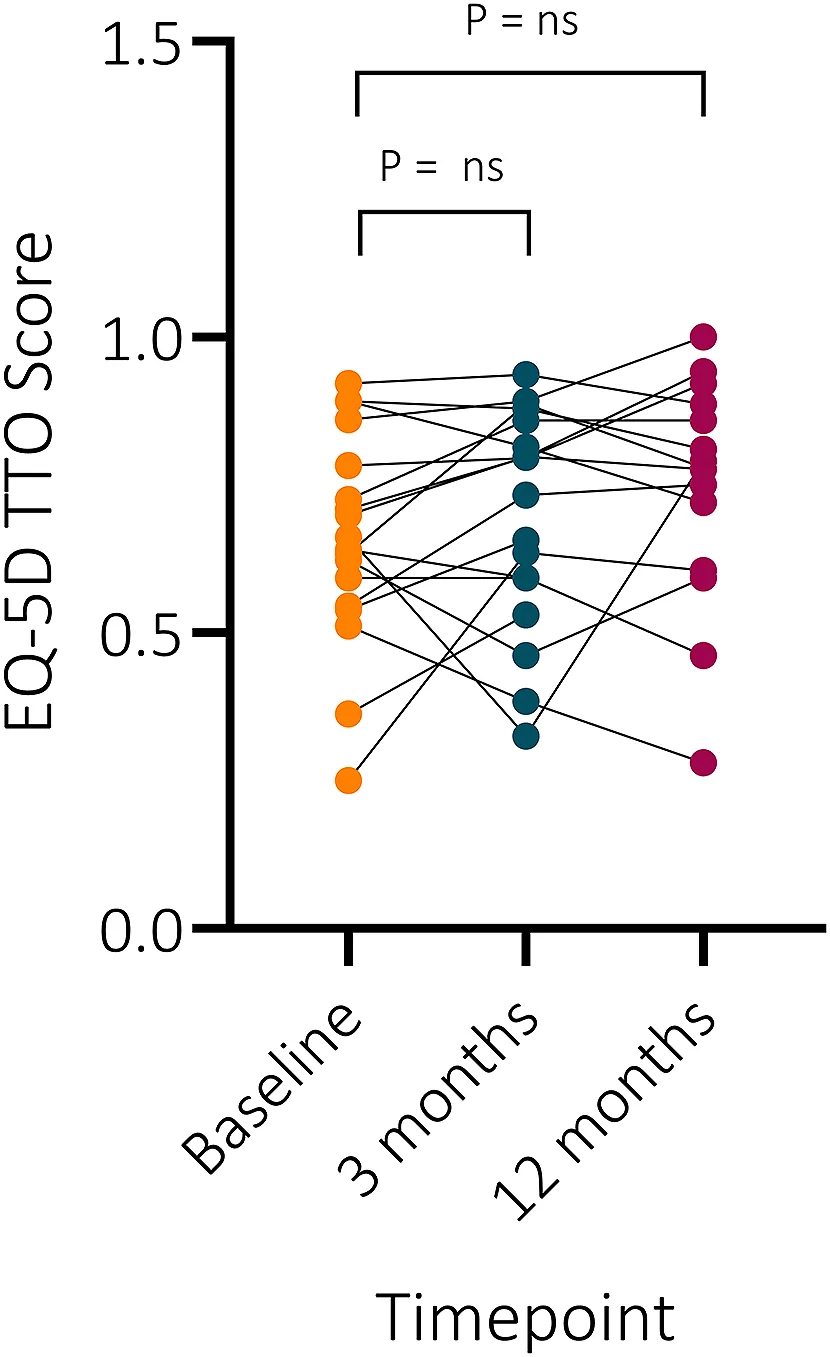
Scatterplot showing EQ-5D-5L TTO scores at baseline, 3 months, and 12 months The mean EQ-5D-5L TTO scores were 0.7 ± 0.2 at baseline and 0.7 ± 0.2 at 3 months (*p* = 0.312, *n* = 18). At 12 months, there was no change in EQ-5D-5L TTO (mean scores were 0.7 ± 0.2 at baseline and 0.7 ± 0.2 at 12 months, *p* = 0.192, *n* = 15). EQ-5D-5L TTO: EQ-5D-5L Time to trade off index score.

**Figure 8:**
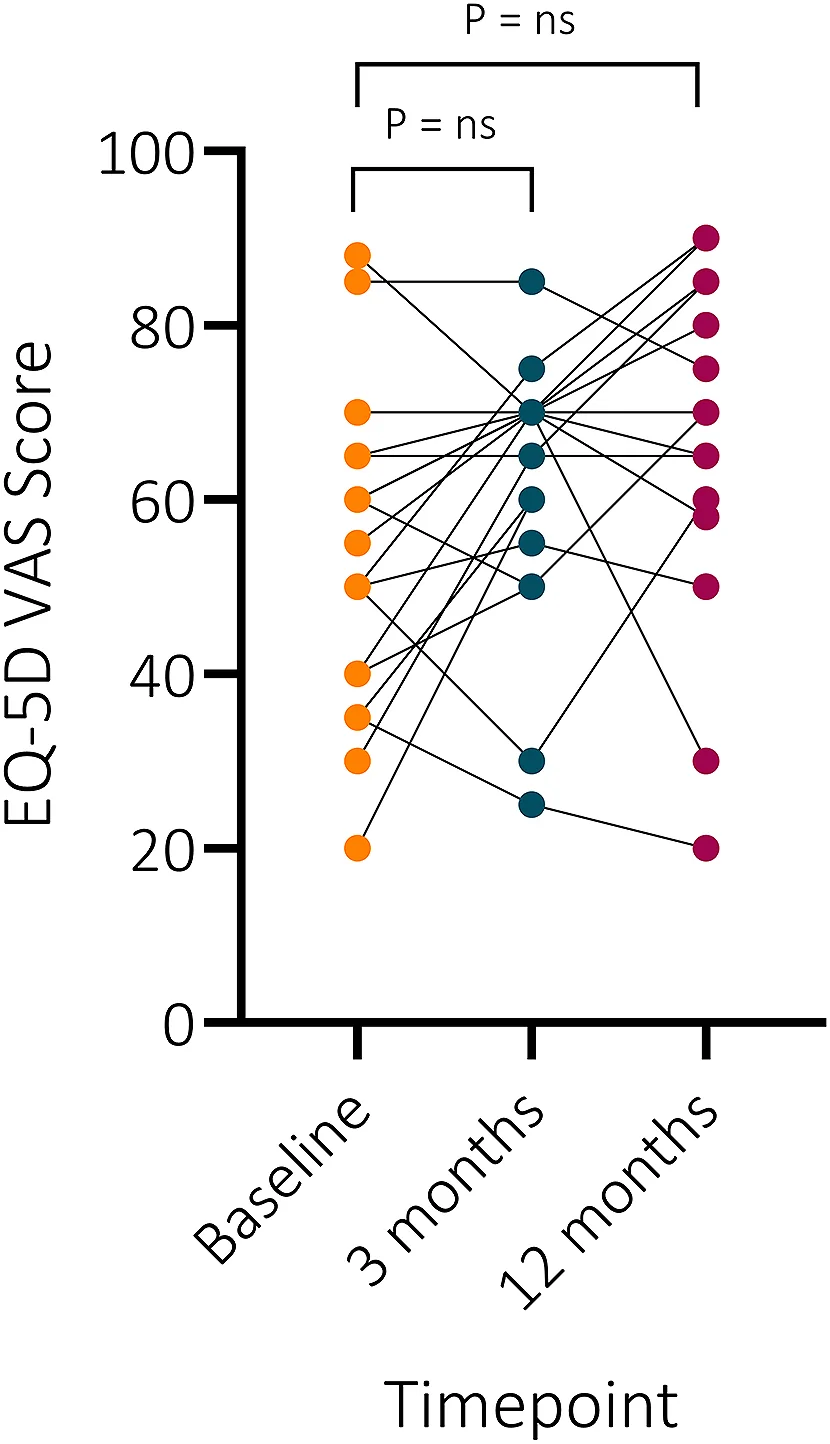
Scatterplot showing EQ-5D-5L Visual Analogue Scores at baseline, 3 months, and 12 months The EQ-5D-5L visual analog scale scores were 53.2 ± 18.2 at baseline and 61.7 ± 15.1 at 3 months (*p* = 0.056, *n* = 18). The EQ-5D-5L visual analog scale scores were 57.5 ± 16.3 at baseline and 66.2 ± 20.6 at 12 months (*p* = 0.251, *n* = 15). EQ-5D-5L VAS: EQ-5D-5L visual analog score.

#### Patient-reported heart failure questionnaire

At 3 months there was a significant improvement in heart failure symptoms as measured by the Minnesota Living with Heart Failure Questionnaire (*n* = 18, 61.9 ± 21.5 to 37.8 ± 23.3; *p* < 0.001; Figure 9). These improvements were maintained at 12 months (*n* = 15, 61.1 ± 22.2 to 38.5 ± 26.7; *p* = 0.003, Figure 9).

**Figure 9:**
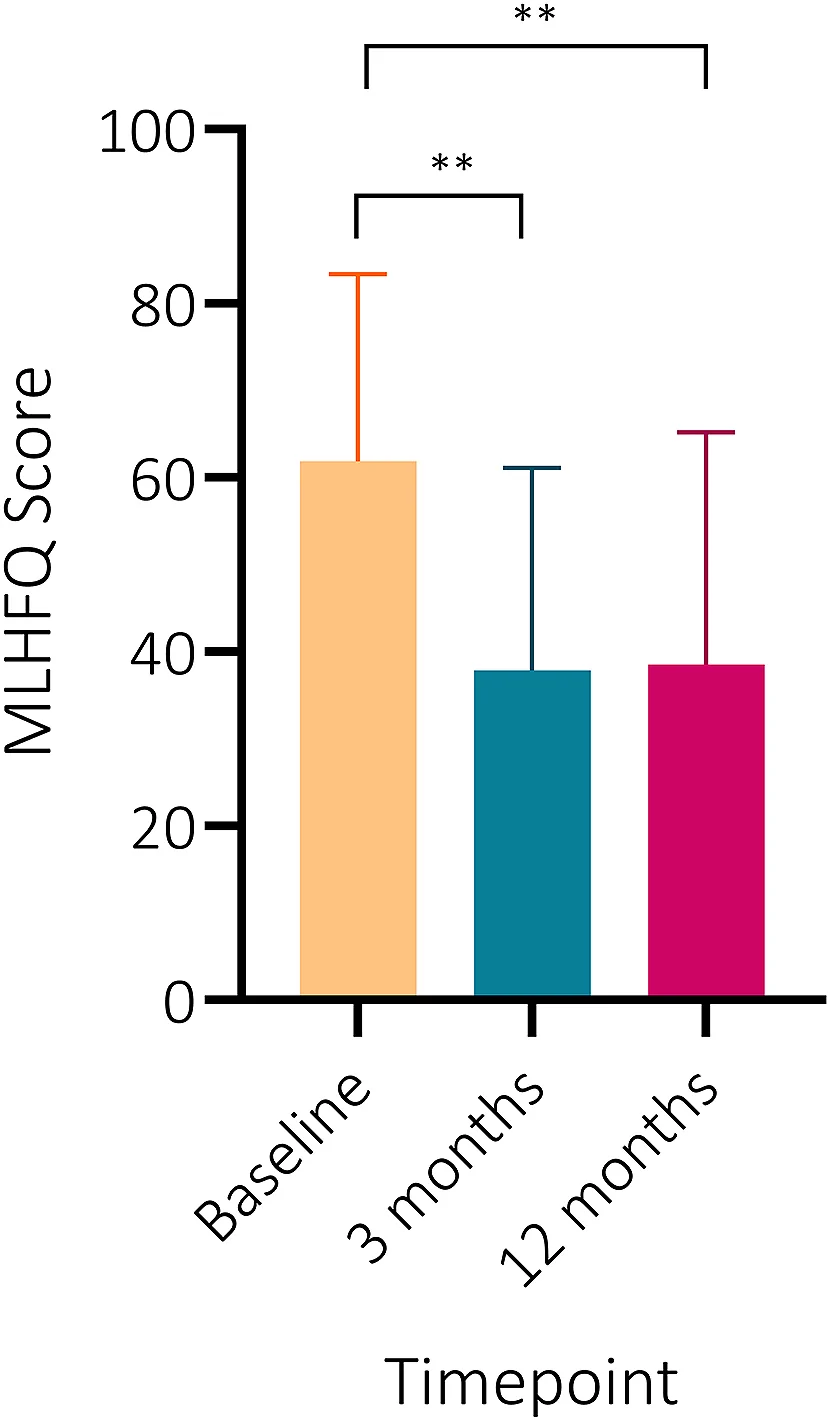
Bar chart showing mean Minnesota Living with Heart Failure Questionnaire scores at baseline, 3 months, and 12 months Participants reported an improvement in symptoms of heart failure at 3 months (61.9 ± 21.5 to 37.8 ± 23.3; *p* < 0.001; *n* = 18). At 12 months, the improvement was maintained compared to baseline (61.1 ± 22.2 to 38.5 ± 26.7; *p* = 0.003; *n* = 15). MLHFQ: Minnesota Living with Heart Failure Questionnaire. *p* values are noted as ∗*p* < 0.05; ∗∗*p* < 0.01. Error bars show standard deviation.

### Exploratory efficacy endpoints

DCM Support was designed as an exploratory, preliminary single-arm feasibility, and safety trial. Although there is no direct control group, pre-specified exploratory efficacy analyses (LVEF and NYHA) were performed by comparing DCM Support with comparator groups from the REGENERATE-DCM trial (NCT01302171; the peripheral saline arm [*n* = 14] and the intracoronary serum arm [*n* = 15]). The results of these exploratory comparisons with historical controls are presented in the supplemental information (Tables S3 and S4).

### Coronary physiology

Exploratory analysis of coronary physiology at procedural baseline demonstrated a mean fractional flow reserve (FFR) of 0.88 ± 0.058, a resting full-cycle ratio (RFR) of 0.9 ± 0.034, a mean coronary flow reserve (CFR) of 2.97 ± 0.44, and a mean index of microvascular resistance (IMR) of 31.93 ± 6.23 which was elevated (i.e., >25). Seven patients (47%) demonstrated microvascular dysfunction (MVD; defined by an IMR >25 and CFR <2.5), with a predominance among females (71% of those with MVD were women). At procedural baseline, higher IMR was associated with lower LVEF (r^2^ = 0.29, *p* = 0.04) and higher NT-proBNP (r^2^ = 0.66, *p* < 0.001). IMR (31.9 ± 24.1; *n* = 15) decreased post-insertion of the Impella device (25.7 ± 29.5, *p* = 0.42) and increased after BMMNC infusion compared to baseline (33.7 ± 37.3, *p* = 0.83, Figure S1).

At 3 months, in participants with MVD (as defined by an IMR >25 and CFR <2.5 at procedural baseline), there was an increase in LVEF (+6.4%, *p* = 0.0469) compared to those with normal microvascular function (1.47%, *p* = 0.5469). Exploratory analysis of coronary physiology also demonstrated that participants with a higher baseline IMR (evidence of microvascular disease) were more likely to see a symptomatic improvement in NYHA class (*p* = 0.0283, r^2^ = 0.3189), alongside an increase in ejection fraction (p = ns, r^2^ = 0.0037) and 6MWT (p = ns, r^2^ = 0.027). A formal between-group comparison of the magnitude of LVEF change (ΔLVEF), or an interaction test, between the MVD and non-MVD groups was not performed given the limited subgroup sizes (*n* = 7 MVD, *n* = 8 non-MVD). These within-group results should therefore be interpreted as hypothesis generating rather than as evidence of a differential treatment effect. Baseline FFR, RFR, and CFR were not tested for independent association with clinical outcomes in regression models, given the limited sample size.

### Inflammatory cytokine analysis

Blood was taken on admission, immediately prior to Impella insertion and cell infusion (in the cardiac catheterization lab and following 5 days of G-CSF infusion), immediately after cell infusion and at 1 day, 3 months, and 12 months post-procedure in a subset of participants to measure changes in inflammatory cytokines in response to the G-CSF pre-treatment as well as the cell infusion procedure (Figure S3). This demonstrated components of both a pro and anti-inflammatory response with inflammatory cytokines increasing (and remaining elevated throughout the procedure) following G-CSF administration prior to Impella insertion and cell infusion (PTX3, s-TREM-1, sCD2, sST2, sTNF-RI, sTNF-RII, and Il-18). The main cytokine change post-Impella insertion and cell infusion was an increase in the anti-inflammatory cytokine soluble receptor for advanced glycation end-products (sRAGE), with the majority of cytokines returning to baseline from day 1 to 12 months post-procedure (Figure S3).

## Discussion

Based on the small sample size, and absence of a control arm, the results of this study should be considered as exploratory and hypothesis generating. This study evaluated the feasibility and safety of combining temporary mechanical circulatory support with intracoronary delivery of autologous BMMNCs in patients with DCM and markedly reduced left ventricular function. The principal finding is that the procedure was well tolerated, with no major periprocedural complications, and was associated with consistent improvements in symptoms and functional capacity sustained over 12 months. As an exploratory phase 2 study, DCM Support was not designed to establish definitive efficacy. A small within-group improvement in LVEF was observed overall, with a numerically greater change in participants with more severe baseline dysfunction, generating hypotheses for future stratified trials.

Regarding the safety of this novel combination delivery technique, the use of the Impella device was not associated with clinical complications during the procedure and led to a potential beneficial decrease in the microvascular resistance (which contrasts to complication rates seen with longer durations of use). Taken in conjunction with the inflammatory cytokine profiling that was performed (which showed an initial increase in inflammatory cytokines during the procedure that decreased at follow up) there was no adverse safety signal related to the cell infusion procedure.

This study provides the first invasive characterization of coronary microvascular function in patients with non-ischemic DCM undergoing intracoronary cell therapy with circulatory support. While the FFR and RFR were in keeping with patients with unobstructed coronary arteries, nearly half of the cohort (47%) exhibited MVD at baseline, supporting the hypothesis that coronary MVD may contribute to progressive myocardial dysfunction and adverse LV remodeling. A sex difference was observed, with 71% of participants with baseline MVD being female, reinforcing known sex-related disparities in coronary microvascular physiology.

Within-group analysis showed an improvement in LVEF at 3 months in participants with baseline MVD (+6.4%, *p* = 0.0469), whereas those with normal baseline microvascular function did not show a significant change (+1.47%, *p* = 0.5469). We did not perform a formal between-group comparison or interaction test of the magnitude of LVEF change given the small subgroup sizes, and therefore these data do not establish that patients with baseline MVD experienced a significantly greater treatment response than those without MVD. Baseline IMR was correlated with NYHA class improvement (*p* = 0.0283, R^2^ = 0.3189), suggesting a possible link between MVD and symptomatic benefit. FFR, RFR, and CFR were not tested for independent association with clinical outcomes given the sample size. These findings support the hypothesis that baseline MVD may be associated with treatment response but do not establish this relationship. Larger studies incorporating formal interaction testing are needed to determine whether elevated baseline IMR/MVD identifies a responsive phenotype that could guide future cell therapy strategies.

Use of the Impella led to a decrease in IMR from baseline which may translate to a beneficial effect on outcome.^18^ Patients with advanced stages of heart failure often experience elevated left ventricular (LV) loading pressures, which create an unfavorable microenvironment that compromises the retention, viability, and growth of transplanted cells.^16^^,^^17^^,^^19^ Introducing circulatory support can help mitigate these harmful conditions by lowering LV-loading pressures. By reducing end-diastolic wall stress and alleviating the mechanical compression exerted on coronary vessels and the microvasculature, percutaneous circulatory assist devices may promote better coronary perfusion, thereby improving cell retention and engraftment.^20^^,^^21^^,^^22^ Interestingly, the IMR increase following cell infusion may have reflected the successful entrapment of cells within the microcirculation.

Analysis of blood taken pre-procedure, post-cell infusion, day 1, 3 months, and 6 months showed a change in a diverse group of cytokines and is summarized in the heatmap (Figure S3). The most striking observation from these exploratory, preliminary results is the 7-fold increase in sRAGE which in animal models has been shown to enhance the reparative capacity of cell therapy.^23^ This, in combination with other cardioprotective effects of sRAGE,^24^ suggests a candidate immunomodulatory pathway warranting further mechanistic investigation.

Although DCM Support did not reach statistical significance for its primary endpoint of change in LVEF, it did demonstrate an improvement in NYHA class (*p* = 0.001), exercise capacity as determined by 6-min walk test (+54.7m, *p* = 0.008) and an improvement in patient-reported symptoms of heart failure (*p* < 0.001) at 3 months. These benefits were maintained at 12 months post-treatment. These results align with prior cell therapy trials in DCM, which have reported improvements in functional capacity and heart failure symptoms, often in the absence of consistent changes in ventricular systolic function.^3^^,^^4^^,^^5^^,^^6^^,^^7^^,^^8^^,^^9^^,^^10^^,^^11^^,^^12^ Interestingly, a recent analysis of cell therapy for heart failure by Bolli et al. demonstrated that, although most cell therapy trials have used LV function as the primary endpoint, growing evidence suggests that cell therapy can impart beneficial effects without necessarily augmenting common indices of LV function.^25^ To date, randomized, placebo-controlled, double-blind trials of cell therapy for heart failure have reported improvements in functional capacity, quality of life, and reduced MACE without detecting a change in LV function. TAC-HFT demonstrated improved functional capacity and quality of life,^26^ CONCERT-HF demonstrated improved quality of life alongside reduced mortality and cardiovascular hospitalization,^27^ ATHENA demonstrated improved quality of life,^28^ and ixCELL-DCM demonstrated reduced mortality and cardiovascular hospitalization^29^; yet none of these trials detected a change in LV function.^25^

Similarly, in DCM Support, looking at a per patient perspective, improvements in NYHA class, exercise tolerance, or quality of life scores did not necessarily correlate with improvements in ejection fraction. Further supporting the notion that clinical and patient-related outcomes can be independent to ejection fraction in identifying new therapies, other studies and risk models have also suggested that predictors of mortality and MACE events in chronic heart failure patients include NYHA class and exercise capacity.^30^^,^^31^^,^^32^^,^^33^ These studies and risk models suggest that the positive results of DCM Support (NYHA class, exercise capacity, and heart failure symptom scores) may lead to improved clinical outcomes (such as cardiovascular death and heart failure hospitalization) despite the study’s failure to meet its primary endpoint of change in ejection fraction and the subjective nature of these endpoints.

In this feasibility and safety study of patients with advanced DCM and severe ventricular dysfunction, intracoronary delivery of autologous bone-marrow cells supported by temporary mechanical unloading was performed safely, with no major procedural complications. Sustained improvements in functional -capacity, symptoms, and quality of life were observed over follow-up; however, the single-arm design means these findings cannot be attributed to mechanical unloading, cell therapy, or their combination, and do not establish that Impella-supported delivery itself confers therapeutic benefit. Causal inference cannot be drawn in the absence of a control group, and these functional and mechanistic signals should be regarded as hypothesis generating. This study demonstrates that combining temporary circulatory support with intracoronary cell delivery is feasible and safe in this high-risk population and supports the design of a randomized controlled trial to determine the incremental value of circulatory support-enabled delivery for biologic and regenerative therapies in advanced non-ischemic cardiomyopathy.

### Limitations of the study

This study has several important limitations. The single-arm design precludes causal inference (i.e., we are unable to ascertain whether the observed findings are related to mechanical unloading, cell therapy, or other factors), thus improvements in functional outcomes must be interpreted cautiously in the absence of a control group and the potential for a placebo effect on subjective endpoints. Ethical concerns relating to sham mechanical support prevented inclusion of a control arm; however, this limits the ability to distinguish treatment effects from regression to the mean or other non-specific influences. The small sample size increases uncertainty around all effect estimates and reduces power to explore predictors of response. Recruitment challenges - including COVID-19-related pauses and the high rate of screen failures due to LVEF thresholds - further limit generalizability. CT-derived LVEF, while reproducible, may differ from echocardiographic measures used in earlier trials, and genetic testing was not systematically performed, an omission that warrants consideration given emerging evidence linking genotype to therapeutic response in DCM. The small sample size also precluded formal interaction testing between MVD subgroups and regression analyses of FFR, RFR, and CFR against clinical outcomes; these should be prioritized in future, larger studies.

Despite these limitations, the study provides a clear demonstration of procedural feasibility and acceptable safety in a high-risk heart failure population. The functional improvements, combined with mechanistic signals from microvascular assessment and inflammatory profiling, suggest that further evaluation in a controlled study is justified. Future trials should incorporate a comparator arm, stratify by genotype, and consider randomization to intracoronary biologic therapy with and without temporary hemodynamic support to determine the incremental value of mechanical unloading.

## Resource availability

### Lead contact

Requests for further information should be directed to and will be fulfilled by the lead contact, Professor Anthony Mathur (a.mathur@qmul.ac.uk).

### Materials availability

Study protocol and statistical analysis plan will be available upon request. This study did not generate new unique reagents.

### Data and code availability

• Data: Individual participant data that underlie the results reported in this article will be available (including text, tables, figures, and appendices) following a data access request to the lead contact, and a data access agreement signed by both parties. The data will be available from 3 months to 36 months after article publication.
• Code: This paper does not report original code.
• Other items: Any additional information required to reanalyze the data reported in this paper are available from the lead contact upon request.

## Acknowledgments

We’d like to thank the staff at Barts Heart Center for their help and work on this study, alongside the members of the DSMB/TSC (Prof. John Martin, Dr Andrew Archbold, Dr Neha Sekhri, and Dr Ollie Guttmann). This work was supported by a grant awarded by the Barts Charity Cardiovascular Programme (MGU0284).

## Author contributions

R.R., investigation and writing – review and editing; M.H., investigation and writing – review and editing; D.P., investigation and writing – review and editing; S.H., investigation and writing – review and editing; M.K., investigation and writing – review and editing; P.P., investigation and writing – review and editing; J.R.W., investigation, resources, and writing – review and editing; T.C.M., investigation and writing – review and editing; A.R., project administration and writing – review and editing; S.B., investigation and writing – review and editing; J.R., project administration, investigation, and writing – review and editing; M.A., project administration, validation, and writing – review and editing; L.W., data curation, validation, and writing – review and editing; C.D., investigation and writing – review and editing; T.K., investigation and writing – review and editing; S.F., investigation and writing – review and editing; J.M., conceptualization and writing – review and editing; C.A., formal analysis and writing – review and editing; D.A.J., supervision, investigation, methodology, and writing – review and editing; A.M., conceptualization, investigation, resources, methodology, writing – original draft, visualization, supervision, and funding acquisition.

## Declaration of interests

A.M. is a board member—Heart Cells Company: company number 10282577; Inventor on US patent no. 11,191,877 - “Magnetised Coronary Stent”; Japanese patent no. 6650930: “Stem Cell Delivery Method”; and UK patent application no. 2406548.4: Method for selection of cells with reparative potential); trustee of the Heart Cells Foundation Charity; received honoraria and a travel bursary from Abbott; received grant awarded by the Barts Charity Cardiovascular Program (MGU0284) which supported this work. J.M. is Inventor on US patent no. 11,191,877 - “Magnetised Coronary Stent”; Japanese patent no. 6650930: ”Stem Cell Delivery Method”); and is a trustee of the Heart Cells Foundation Charity. T.R.K. is an advisory board member for Abiomed and Abbott vascular. C.A. has received consulting fees from Queen Mary University of London. All other authors have nothing to declare.

## Declaration of generative AI and AI-assisted technologies in the writing process

During the preparation of this work the author(s) used ChatGPT in order to proof-read the text. After using this tool/service, the author(s) reviewed and edited the content as needed and take(s) full responsibility for the content of the publication.

## STAR★Methods

### Key resources table

**Table:** 

REAGENT or RESOURCE	SOURCE	IDENTIFIER
**Software and algorithms**		

Medis CT Suite 4.0	Medis Medical Imaging Systems B.V., Leiden, The Netherlands	https://medisimaging.com/software-solutions/medis-suite-ct/
STATA Statistical software, version 16	StataCorp LLC, Texas	https://www.stata.com/stata16/

**Other**		

Lymphoprep	Axis Shield	1114545
Filgrastim	Zarzio, Sandoz Ltd. Surrey, United Kingdom	https://bnf.nice.org.uk/drugs/filgrastim/
Impella CP device	Abiomed, Danvers MA	https://www.jnjmedtech.com/en-US/products/cardiovascular/impella-heart-pumps/impella-cp-with-smartassist/
Pressure Wire X & Coroventis software	Abbott, Boston, USA	https://www.cardiovascular.abbott/us/en/hcp/products/percutaneous-coronary-intervention/coroventis-coroflow/about.html

### Experimental model and study participant details

DCM Support was a prospective, single-centre, single-arm exploratory clinical study conducted at Barts Heart Centre, London, United Kingdom. Adult participants aged 18-85 years with non-ischemic dilated cardiomyopathy (DCM), left ventricular ejection fraction (LVEF) ≤35%, and persistent symptoms despite at least three months of guideline-directed medical therapy were eligible for enrolment. Key exclusion criteria included clinically significant valvular disease, reversible cardiomyopathy, active infection, contraindications to bone-marrow aspiration or femoral large-bore access, left-ventricular thrombus, severe comorbidity limiting 1-year survival and inability to complete follow-up. Full criteria are presented in Table 1.

Among participants included in the primary analysis, the mean age was 54.0 ± 13.8 years. Eleven participants (57.9%) were male and eight (42.1%) were female. Ethnicity was White British (78.9%), South Asian (5.3%), Asian Other (5.3%), Black Caribbean (5.3%) and Black Other (5.3%). Socioeconomic status was not collected as part of the study protocol. Gender identity, ancestry, and other demographic variables beyond ethnicity were not recorded. Although sex was recorded and reported descriptively, the study was not powered to perform formal sex-based analyses and gender identity data were not collected. Consequently, we were unable to evaluate whether procedural safety, functional outcomes, or mechanistic responses differed according to sex or gender. Given known sex-related differences in heart failure presentation, coronary microvascular function, and dilated cardiomyopathy phenotype, the absence of adequately powered sex- and gender-based analyses limits the generalizability of these findings.

#### Ethics statement

The study was conducted in accordance with the Declaration of Helsinki and was approved by an NHS Research Ethics Committee (REC number: 18/SC/0195). All participants provided written informed consent before undergoing any study-specific procedures. Study data were collected and managed using REDCap electronic data capture tools hosted at Queen Mary University of London.

### Method details

#### Study design and primary and secondary outcomes

DCM Support was a prospective, single-centre, single-arm exploratory study conducted at Barts Heart Centre (London, UK). The protocol has been previously published [18]. The design follows CONSORT guidelines for pilot studies and was registered at ClinicalTrials.gov (NCT03572660). Trial flow is depicted in Figure 1. The objective was to evaluate the safety and feasibility of intracoronary autologous bone-marrow cell delivery using temporary mechanical circulatory support in patients with severe dilated cardiomyopathy (DCM).

Following informed consent and confirmation of eligibility, enrolled participants received 5 days of weight adjusted (10 mcg/kg/day) subcutaneous granulocyte colony-stimulating factor (G-CSF; Filgrastim [Zarzio], Sandoz Ltd. Surrey, United Kingdom) therapy. On day 5, 54mls of bone marrow was harvested from the posterior iliac crest and processed using the open Ficoll technique at a specialised, GCP-compliant, MHRA-regulated laboratory. The bone marrow-derived mononuclear cells (BMMNCs) were then suspended in 10mL of normal saline and returned to the hospital later that day. In the cardiac catheterization laboratory, access was obtained under ultrasound guidance, and the Impella CP device was inserted via large-bore femoral arterial access. The intracoronary cell infusion technique has been previously described.^1^ In brief, once the Impella device had been sited and continuous flow established, the BMMNCs were infused into the coronary arteries via radial access using the ‘stop-flow’ technique (with the aim to infuse >90% into the arteries supplying the left ventricle). Coronary stasis was achieved via inflation of a rapid exchange balloon catheter, and 3.3.mls of BMMNCs were infused over a 3-minute period through a microcatheter with its tip distal to the balloon. This process was repeated 3 times until all 10mls of cells were infused. Once the cells were delivered, the Impella’s flow rate was gradually decreased over 5 minutes, and it was removed in the catheterization laboratory. The femoral artery puncture was closed using the Manta® vascular closure device (Essential Medical, Inc, Pennsylvania, USA) in 11 participants as per the manufacturer’s guidance. In 9 participants, femoral access was closed using a combination of Perclose ProGlide (Abbott Vascular, California, USA) and an Angio-Seal® VIP Vascular Closure Device (Terumo, Tokyo, Japan). The angioplasty access was closed as per local protocol. Dependent on their clinical condition, participants were discharged the next day after vascular access site assessment. Participants were followed up via telephone at 1 month post-procedure, and in-hospital at 3 and 12 months.

The trial’s primary endpoint was the change in LVEF at 3 months from baseline as measured by a cardiac computed tomography scan (CT). Secondary safety endpoints included procedural morbidity and mortality, alongside major adverse cardiac events (MACE; defined as: all-cause death, myocardial infarction, hospitalisation for heart failure and major arrhythmias) and the incidence of stroke at 3 and 12 months. Secondary efficacy endpoints at 3 months were change from baseline in: NYHA class, NT-proBNP levels, exercise capacity (as measured by the 6-minute walk test), patient-reported heart failure symptoms as measured by the Minnesota Living with Heart Failure Questionnaire (MLHFQ), and quality of life scores as measured by the EQ-5D-5L questionnaire.

#### Cardiac computed tomography (CT) for the assessment of LVEF

LVEF assessment was undertaken using CT as a single imaging modality was preferable for uniformity. CT has comparable reproducibility compared to MRI,^34^^,^^35^^,^^36^ even in patients with reduced EF,^35^ and cardiac devices preclude participants from undergoing a research cardiac MR scan as per our local protocol (of the 20 participants treated per protocol, 9 had an implantable cardioverter-defibrillator, 2 had an implantable cardiac resynchronisation therapy pacemaker and 4 had a cardiac resynchronization therapy defibrillator). Furthermore, to de-risk the invasive procedure, and based on our previous experience, patients underwent a procedural planning CT scan to assess coronary anatomy and vascular access (to ensure safe Impella insertion) and therefore it was minimal extra radiation, time and contrast to provide an assessment of LVEF.

All trial CTs were performed using a third-generation dual-source CT scanner (Somatom FORCE, Siemens). Multi-phase cardiac datasets with full left ventricular coverage were acquired using standard protocols. The scans were anonymized and underwent blinded analysis using Medis CT Suite 4.0 (Medis Medical Imaging Systems B.V., Leiden, The Netherlands) by two experienced operators. For full details of CT-imaging and analysis protocols see supplemental information: Appendix 1 and 2.

#### Assessment of coronary physiology

Invasive evaluation of coronary microvascular function was conducted in a predefined subgroup of fifteen participants as part of the study’s exploratory mechanistic analysis. Measurements of resting flow reserve (RFR), fractional flow reserve (FFR), coronary flow reserve (CFR), and the index of microvascular resistance (IMR) were obtained using the bolus thermodilution technique in the left anterior descending artery (LAD). These parameters were assessed at three distinct time points during the intracoronary cell infusion procedure: (1) at baseline, prior to device insertion; (2) after deployment of the Impella CP device and escalation to maximal flow support; and (3) immediately following cell infusion into the LAD.

#### Assessment of mediators of inflammation

In addition, a subset of participants underwent serial blood sampling for exploratory analysis of circulating pro-inflammatory cytokines and angiogenic factors. Samples were collected at six time points throughout the study: (1) at baseline (upon admission), (2) following completion of the G-CSF injection regimen and immediately prior to cell infusion, (3) immediately after cell infusion, and subsequently at (4) 1 day, (5) 3 months, and (6) 12 months post-infusion. The panel of cytokines and growth factors measured is detailed in Figure S3.

### Quantification and statistical analysis

#### Sample size and statistical analysis

The sample size was informed by variance estimates from REGENERATE-DCM and intended to support exploratory evaluation of ventricular function alongside feasibility and safety outcomes. This equated to a sample size of 17 patients which was increased to 20 patients to account for 15% lost to follow-up. The REGENERATE-DCM trial showed an absolute improvement in LVEF of 5.37% ± 7.39% at 3 months in the cell therapy arm.^3^ The sample size calculations were performed using Stata statistical software, version 16 (StataCorp LLC, Texas). The safety population for the study consists of all patients who were administered the first dose of G-CSF (*n* = 23). The full analysis population consists of all patients wherein it is possible to measure the primary endpoint (i.e. those who received the full protocol intervention [the full G-CSF course and underwent cell administration] and who had a valid LVEF measurement at baseline and 3 months) (*n* = 19). Baseline demographic and clinical characteristics were summarised using mean and standard deviation for continuous variables and frequency and percentage for categorical variables. The median and interquartile range was used to further summarise NT-proBNP due to the skewed distribution of measurements.

Differences between the month 12, month 3 and baseline measurements for continuous variables were assessed using the paired t-test to calculate the mean difference, 95% confidence interval and *p*-value. The exception was NT-proBNP where a paired sign rank test was used to compare the month 12, month 3 and baseline outcomes due to the skewed distribution of measurements. Changes in NHYA classification were also assessed using the paired sign rank test. The occurrence of safety events was summarised by the frequency and percentage of patients experiencing a safety event and the frequency and percentage of each safety event. Comparisons between pre-specified subgroups used the independent two-sample t-test to evaluate differences in the baseline to month 3 change in LVEF between the subgroups. The exploratory microvascular dysfunction analysis assessed changes from baseline to the clinical assessment points using the paired t-test. The number, percentage and associated 95% confidence intervals for patients assessed as meeting the criteria for microvascular dysfunction was also calculated. An exploratory comparative analysis between patients in DCM SUPPORT and those in the peripheral saline and intracoronary serum arms of the REGENERATE-DCM trial used a linear regression model adjusted for baseline LVEF to assess differences in changes to LVEF. Changes in inflammatory cytokines are presented as a heat map. *p* values are noted as ∗*p* < 0.05; ∗∗*p* < 0.01.

### Additional resources

ClinicalTrials.gov: NCT03572660.
