# Supplementary material for: Procedural circulatory support-enabled intracoronary cell delivery in advanced dilated cardiomyopathy
Source: iScience. 2026 Sep 23;29(10):117543. doi: 10.1016/j.isci.2026.117543 (PMC13634339; doi:10.1016/j.isci.2026.117543)
Supplement: Supplementary file 1 [file mmc1.pdf]

iScience, Volume *29*

Supplemental information

Procedural circulatory support-enabled

intracoronary cell delivery

in advanced dilated cardiomyopathy

Rohini Ramaseshan, Mohsin Hussain, Dhanuka Perera, Stephen Hamshere, Matthew Kelham, Pedro Pinto, James R. Whiteford, Thomas C. Moseley, Alice Reid, Sonia Bastos, Jessry Russell, Mervyn Andiapen, Lucinda Wynne, Ceri Davies, Thomas Keeble, Samer Fawaz, John Martin, Cono Ariti, Daniel A. Jones, and Anthony Mathur

Supplementary Material

Appendix 1: Cardiac CT protocol	1
Appendix 2: Cardiac CT analysis	2
Table S1: LV analysis at 3 months	3
Table S2: Analysis of ATIMP cell counts vs change in LVEF at 3 months	3
Table S3: Exploratory comparative analysis: Left ventricular ejection fraction (%)	4
Table S4: Exploratory comparative analysis: Change in NYHA classification	4
Table S5: Subgroup analysis: Change in LVEF (%) in selected subgroups	5
Figure S1: Change in IMR	6
Figure S2: Correlation between change in IMR and LVEF	6
Figure S3: Heatmap showing the relative levels of pro angiogenic and inflammatory markers in participant serum.	7

Appendix 1: Cardiac CT Protocol

All trial CTs were performed using a third-generation dual-source CT scanner (Somatom FORCE, Siemens). Multi-phase cardiac datasets with full left ventricular coverage were acquired using standard protocols. All scans were performed in the craniocaudal direction. An initial scouting x-ray (topogram) was performed to ensure correct patient alignment for the remainder of the scan. The scan was initiated when contrast arrived within the ascending aorta using a small volume test bolus. A total of 50 - 100 mls of contrast was used for the scan with a duration of 10 – 15 minutes for the scan. Standard reconstruction algorithms were used to convert the raw data into interpretable images, which was then analysed on specialist software.

Appendix 2: Cardiac CT Analysis Standard

CTs were analysed by two independent blinded clinicians with experience in reporting LVEF by CT. Once uploaded into the Medis viewer, anonymised CT images were reformatted into a short axis stack covering the entire myocardium. All reconstructions were formatted into 10mm thick slices, applied to all timepoints. Medis QMass software was then used for LVEF analysis. ED and ES were manually selected and endocardial and epicardial contours were drawn for each slice. Once the report was created with the resultant LV volume and LVEF, the average of the 2 independently reported EFs was taken as the final result. Any results with a discrepancy of > +/- 10% of the absolute LVEF measurement (e.g., if LVEF measured at 45% - anything between 40% and 50% was accepted) were independently reviewed and adjudicated by a third clinician (independent blinded imaging consultant). The adjudicated result was the average of the imaging consultant's result and the reviewer's value that is within 10% of the imaging consultant's result. All adjudicated results were considered final.

Table S1: LV Analysis at 3 months

	N	Baseline Mean	Baseline (SD)	Month 3 Mean	Month 3 (SD)	Mean Difference	95% CI	P
**Primary Endpoint**								
Left ventricle ejection fraction (%)	19	24.9	(10.1)	27.8	(10.4)	2.9	(-0.3, 6.1)	0.074
**Secondary Endpoint**								
Left ventricular end-diastolic volume (mL)	19	328.8	(84.9)	336.2	(83.8)	7.4	(-14.1, 28.9)	0.480
Left ventricular mass (g)	19	164.7	(57.4)	168.3	(51.9)	3.6	(-7.6, 14.8)	0.511
Left ventricular end-systolic volume (mL)	19	250.3	(81.3)	248.3	(85.6)	-2	(-18.1, 14.1)	0.793

Table S2: Analysis of ATIMP cell counts vs change in LVEF at 3 months

	Cell counts			Mean change in LVEF (%)		
	N	Mean	(SD)	Mean Difference	95% CI	P
**Analysis of cell counts**						
Absolute CD 34^+ cell number x10^6	11	4.0	3.9	0.6	(-0.1, 1.2)	0.092
Total WBC Count x10^6	19	298.9	102.5	0.0	(-0.0, 0.0)	0.285

P values from linear regression model adjusted for baseline LVEF.

Note: Mean difference is mean change in LVEF % per unit change in the cell count variable.

Table S3: Exploratory comparative analysis: Left ventricular ejection fraction (%)

	Change in Left ventricular ejection fraction (%) baseline to month 3											
	Baseline			Month 3			Month 3 - Baseline			DCM SUPPORT - comparator		
	N	Mean	(SD)	N	Mean	(SD)	N	Difference*	95% CI	Difference **	95% CI	P
**Study population**												
DCM Support	19	24.9	(10.1)	19	27.8	(10.4)	19	2.9	(-0.3, 6.1)			0.377
REGENERATE DCM Peripheral Saline	14	29.8	(8.9)	14	28.7	(8.7)	14	-1.1	(-5.8, 3.6)	2.1	(-2.2, 6.5)	
REGENERATE DCM Intracoronary serum	15	41.7	(15.3)	15	40.6	(12.8)	15	-1.1	(-4.2, 2.0)	-1.0	(-5.9, 4.0)	

CI = confidence interval; P value from linear regression model adjusted for baseline LVEF.

*Mean difference from paired t-test

**Difference is the mean change from baseline in DCM SUPPORT minus the mean change from baseline of the comparator, adjusted for baseline LVEF

Table S4: Exploratory comparative analysis: Change in NYHA classification

	DCM SUPPORT		DCM Peripheral Saline			DCM Intracoronary Serum		
	Baseline to Month 3		Baseline to Month 3		DCM SUPPORT v REGENERATE DCM Peripheral Saline	Baseline to Month 3		DCM SUPPORT v REGENERATE DCM Intracoronary Serum
	N	(%)	(N)	(%)	P	(N)	(%)	P
**Change in NYHA class**								
**Improved**	11	(61.1)	0	(0.0)	<0.001	1	(6.7)	<0.001
**No change**	7	(38.8)	13	(92.9)		12	(80.0)	
**Worse**	0	(0)	1	(7.1)		2	(13.3)	

NYHA = New York Heart Association Functional Classification; P values from Wilcoxon-Rank-Sum test.

Table S5: Subgroup analysis: Change in LVEF (%) in selected subgroups

		**Baseline**		**Month 3**		Mean		**Difference in subgroups**		
**Subgroups**	N	Mean	(SD)	Mean	(SD)	Difference	95% CI	Difference	95% CI	P
**Age category (years)**										
<= 50	8	30.8	(9.7)	31.6	(10.2)	0.8	(-6.0, 7.6)			
50+	11	20.5	(8.3)	25.0	(10.2)	4.4	(0.9, 7.9)	-3.6	(-10.1, 2.9)	0.251
**Gender**										
Male	11	25.3	(10.4)	26.4	(10.9)	1.1	(-4.3, 6.5)			
Female	8	24.2	(10.3)	29.7	(10.2)	5.4	(2.8, 8.0)	-4.4	(-10.7, 1.9)	0.164
**Smoking status**										
Never	12	26.4	(10.3)	30.7	(10.2)	4.3	(-0.1, 8.7)			
Current or ex	7	22.2	(10.0)	22.8	(9.6)	0.6	(-5.0, 6.2)	3.7	(-2.9, 10.3)	0.257
**Troponin T (ng/L)**										
<=20	11	29.3	(10.4)	32.4	(10.7)	3.0	(-2.3, 8.3)			
20+	8	18.7	(5.8)	21.5	(6.2)	2.7	(-1.4, 6.8)	0.3	(-6.4, 7.0)	0.930

LVEF = Left ventricle ejection fraction

P values from t-test comparing differences between subgroups

Figure S1:

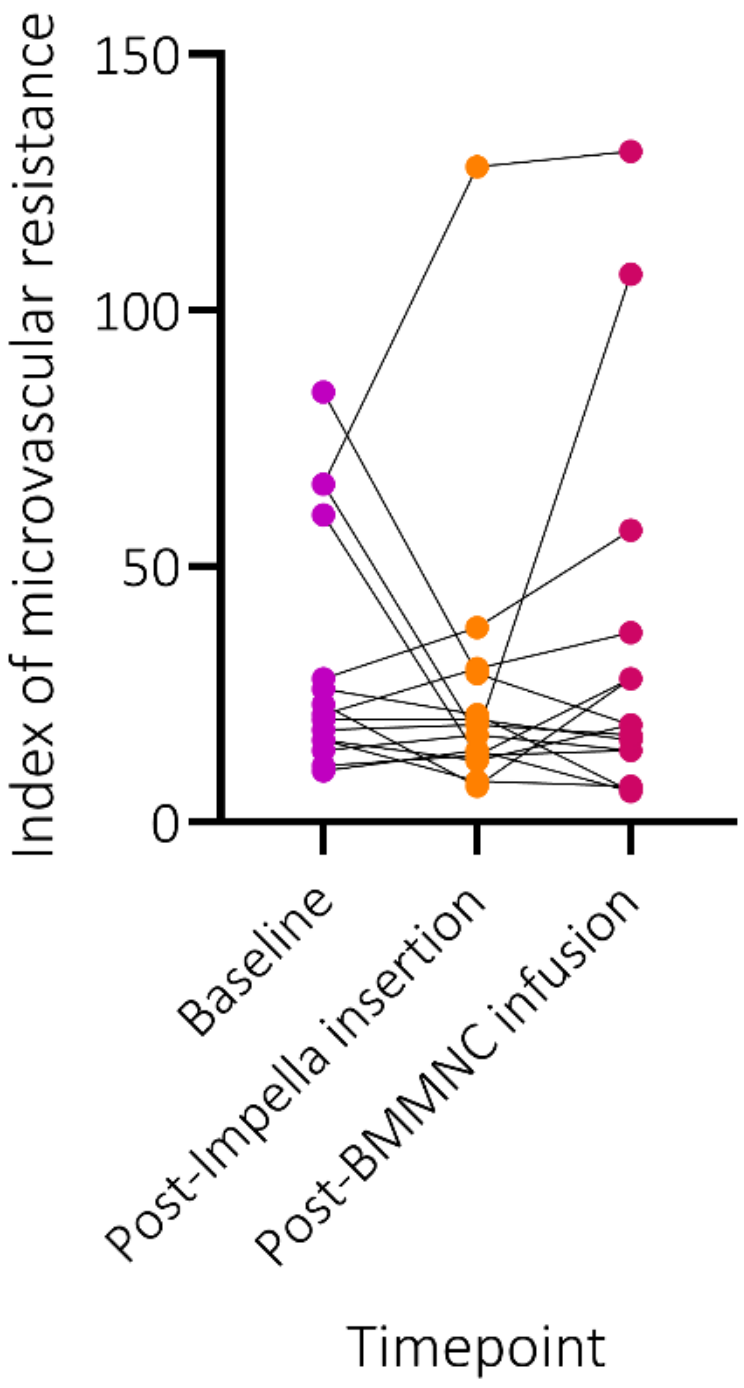

Figure S1: Change in IMR in 15 participants. Baseline IMR was 31.9 ± 24.1. IMR decreased post insertion of the Impella device compared to baseline (25.7 ± 29.5, p = 0.42) and increased after BMMNC infusion compared to baseline (33.7 ± 37.3, P = 0.83).

BMMNC: Bone marrow-derived mononuclear cells; IMR: Index of microvascular resistance

Figure S2:

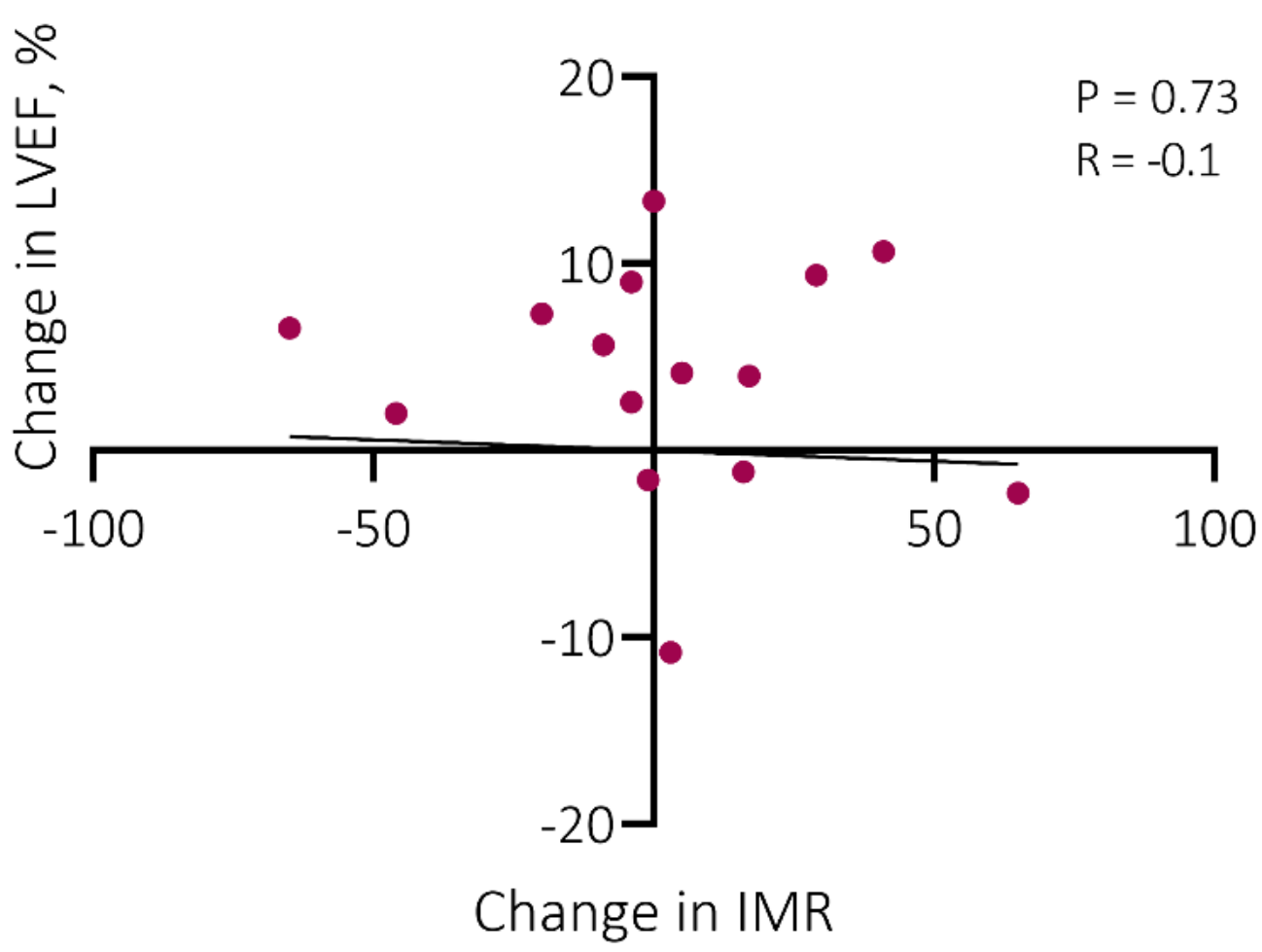

Figure S2: Dot plot showing correlation between the change in IMR from baseline to post BMMNC infusion & change in LVEF from baseline to 3 months (P = 0.73, r^2 = -0.1).

BMMNC: Bone marrow-derived mononuclear cells; IMR: Index of microvascular resistance; LVEF: Left ventricular ejection fraction

Figure S3:

	Baseline	Pre	Post	Day 1	3 month	12 month	
VEGFA	1.00	1.15	0.01	0.62	0.96	0.76	Angiogenesis
VEGFD	1.00	0.58	0.41	0.47	0.99	0.89	Angiogenesis
sCD40L	1.00	0.77	0.02	0.01	0.60	1.22	Clotting
sRAGE	1.00	0.6	7.0	0.6	1.1	1.3	Anti-inflammatory
PTX3	1.00	3.49	3.31	1.38	0.52	1.57	Inflammation
TGFbeta	1.00	0.3	0.2	0.0	2.4	1.4	Ant-inflammatory
PAI-1	1.00	1.02	0.42	0.77	1.24	1.06	Clotting
sTREM-1	1.00	1.81	1.41	1.29	1.65	1.31	Inflammation
sCD25	1.00	2.08	1.85	2.02	1.32	1.53	Inflammation
sST2	1.00	1.26	1.17	1.00	1.23	1.29	Inlfammation
sTNF-RI	1.00	2.65	1.73	1.62	1.59	1.65	Anti-inflammatory
sTNF-RII	1.00	2.44	2.12	2.17	1.42	1.89	Anti-inflammatory
CX3CL1	1.00	1.04	0.99	1.00	1.20	1.16	Inflammation
sCD130	1.00	0.82	0.68	0.84	1.72	1.56	Inflammation
MCP-1	1.00	0.60	0.39	0.81	1.25	0.99	Inflammation
IL-18	1.00	1.71	1.90	2.16	1.45	1.03	Inflammation
Il-1beta	1.00	0.99	1.04	1.33	1.31	1.29	Inflammation
IFN-alpha-2	1.00	0.94	1.03	0.71	1.39	1.49	Inflammation
IFN-gamma	1.00	1.01	1.05	1.23	1.20	1.29	Inflammation
TNFalpha	1.00	1.21	1.01	1.11	1.22	1.30	Inflammation
Il-33	1.00	1.06	1.04	1.31	1.30	1.39	Inflammation
Il-10	ND	ND	ND	ND	ND	ND	Inflammation
IFN-α2	ND	ND	ND	ND	ND	ND	Inflammation
Il-12p70	ND	ND	ND	ND	ND	ND	Inflammation
Il-17A	ND	ND	ND	ND	ND	ND	Inflammation

Fold change from baseline: 7.0 / 1.0 / 0.0

Figure S3: Heatmap showing the relative levels of pro angiogenic and inflammatory markers in patient serum. Baseline samples were taken on admission, followed by samples taken after the G-CSF injection regimen and immediately prior to the infusion procedure(Pre), directly after the intracoronary cell infusion (Post), followed by samples 1 day, 3 months and 12 months post the procedure.

VEGFA and D levels were measured by ELISA and the remaining markers were assayed using Legendplex bead arrays. Fold change was calculated using the baseline values for each marker to normalise the data. The heatmap was generated in Microsoft Excel. (N=14 for VEGFA and D, and n=7 for the remaining markers).
